# No long-term effects of antenatal synthetic glucocorticoid exposure on epigenetic regulation of stress-related genes

**DOI:** 10.1038/s41398-022-01828-x

**Published:** 2022-02-16

**Authors:** Svenja Müller, Dirk Moser, Leonard Frach, Pauline Wimberger, Katharina Nitzsche, Shu-Chen Li, Clemens Kirschbaum, Nina Alexander

**Affiliations:** 1 Department of Genetic Psychology, Faculty of Psychology, Ruhr Universität Bochum, Universitätsstr. 150, 44801 Bochum, Germany; 2 Department of Clinical, Educational and Health Psychology, Division of Psychology and Language Sciences, University College London, 26 Bedford Way, London, WC1H 0AP UK; 3 Department of Gynecology and Obstetrics, Medical Faculty and University Hospital Carl Gustav Carus, Technische Universität Dresden, Fetscherstr. 74, 01307 Dresden, Germany; 4 Faculty of Psychology, Technische Universität Dresden, Zellescher Weg 17, 01602 Dresden, Germany; 5 CeTI – Centre for Tactile Internet with Human-in-the-Loop, Technische Universität Dresden, Georg-Schumann-Str. 9, 01187 Dresden, Germany; 6 Department of Psychiatry and Psychotherapy, Philipps University Marburg, Rudolf-Bultmann-Str. 8, 35039 Marburg, Germany; 7 Center for Mind, Brain and Behavior, Philipps University Marburg, Hans-Meerwein-Str. 6, 35032 Marburg, Germany

**Keywords:** Clinical genetics, Physiology

## Abstract

Antenatal synthetic glucocorticoid (sGC) treatment is a potent modifier of the hypothalamic-pituitary-adrenal (HPA) axis. In this context, epigenetic modifications are discussed as potential regulators explaining how prenatal exposure to GCs might translate into persistent changes of HPA axis “functioning”. The purpose of this study was to investigate whether DNA methylation and gene expression profiles of stress-associated genes (*NR3C1; FKBP5; SLC6A4)* may mediate the persistent effects of sGC on cortisol stress reactivity that have been previously observed. In addition, hair cortisol concentrations (hairC) were investigated as a valid biomarker of long-term HPA axis activity. This cross-sectional study comprised 108 term-born children and adolescents, including individuals with antenatal GC treatment and controls. From whole blood, DNA methylation was analyzed by targeted deep bisulfite sequencing. Relative mRNA expression was determined by RT-qPCR experiments and qBase analysis. Acute stress reactivity was assessed by the Trier Social Stress Test (TSST) measuring salivary cortisol by ELISA and hairC concentrations were determined from hair samples by liquid chromatography coupled with tandem mass spectrometry. First, no differences in DNA methylation and mRNA expression levels of the stress-associated genes between individuals treated with antenatal sGC compared to controls were found. Second, DNA methylation and mRNA expression levels were neither associated with cortisol stress reactivity nor with hairC. These findings do not corroborate the belief that DNA methylation and mRNA expression profiles of stress-associated genes (*NR3C1; FKBP5; SLC6A4)* play a key mediating role of the persistent effects of sGC on HPA axis functioning.

## Introduction

Prenatal maternal stress and exposure to antenatal synthetic glucocorticoids (sGC) can have long-term consequences on major stress response systems and mental health [1, 2]. Importantly, women at risk for preterm delivery are routinely treated with sGCs such as betamethasone (BETA) and dexamethasone (DEX) which directly cross the placenta and promote fetal lung maturation [3]. Apart from their clear benefits, several studies have identified antenatal sGC exposure as a potent modifier of the hypothalamus-pituitary-adrenal (HPA) axis (for a review see [1]), possibly leading to an increased risk for stress-related disorders later in life [2]. For example, our group observed increased cortisol stress responses in sGC-treated term-born children compared with untreated controls [4] that persisted into late adolescence [5].

Epigenetic modifications such as DNA methylation (DNA_M_) are discussed as a central mechanism explaining how in utero exposure to GCs might translate into persistent changes of HPA axis functioning [6, 7]. In the promoter region, together with histone modifications DNA_M_ serves as a transcriptional regulator, e.g., by influencing transcription factors from binding to regulatory elements [7]. There is now considerable evidence that the fetal epigenome is responsive to a broad range of intrauterine environmental exposures, including prenatal maternal stress and in utero sGC exposure [8]. To date, the majority of findings derive from candidate gene studies investigating DNA_M_ states at genes involved in neurotransmitter (*SLC6A4* [9]) and stress hormone regulation (*NR3C1* [10]) The most prominent example involves the glucocorticoid receptor gene (*NR3C1)*, where different forms of prenatal stress have been associated with increased DNA_M_ at one single CpG site (meta-analysis [11]). Given the pivotal role of glucocorticoid receptor (GR) signaling in negative feedback regulation of the HPA-axis, respective epigenetic changes may account for permanently altered glucocorticoid levels as it has been suggested by landmark rodent studies [12]. Another candidate region constitutes the *FK506 binding protein 5* (*FKBP5*) gene that also acts as an important modulator of the HPA axis. FKBP5 provides an ultrashort negative feedback loop for GR signaling by reducing its cortisol binding affinity and impeding translocation of the receptor complex to the nucleus [13]. Several studies reported associations between early adversity (e.g., childhood trauma, institutionalized care) and a demethylation of a glucocorticoid response element (GRE) located in intron 7 of the *FKBP5* gene (e.g. Klengel et al. [14]), although not without inconsistences (e.g. Alexander et al. [15]). In turn, this epigenetically-induced upregulation of *FKBP5* presumably leads to a persistent GR resistance and a disruption of the HPA-axis feedback control [16]. First studies on prenatal stress exposures such as maternal affective disorders [17] and perceived distress [18] have also produced mixed findings by investigating a range of different CpG sites within the *FKBP5* gene. Another stress-related gene that has also received considerable attention in epigenetic studies on early adversity (for a review see [9]) and HPA-axis functioning (e.g. refs. [19, 20]) is the serotonin transporter gene (*SLC6A4)*. Regarding prenatal stress, one study revealed a negative association of maternal depressive mood during pregnancy and *SLC6A4* DNA_M_ levels [21] while other studies reported no effects of maternal prenatal stressors (e.g. Wankerl et al. [22]). Given the pivotal impact of serotonergic signaling on HPA-axis regulation [23], *SLC6A4* DNA_M_ profile may well account for long-term changes in cortisol output.

Only few studies have so far investigated epigenetic correlates of prenatal GC exposure, although GC reflects a key mechanism of how prenatal stress translates into persistent DNA_M_ changes [24]. In an elegant model of human hippocampal progenitor cell (HPC) lines, GC exposure was found to induce widespread changes of DNA_M_, with the most pronounced effects observed during the proliferation and differentiation stage [25]. Regarding stress-related candidate genes in specific, together with another set of in vitro studies a demethylation of *FKBP5* intron 7 in HPC cell lines following DEX treatment was demonstrated [14, 25]. While evidence from living humans is still rare, a first genome-wide association study identified 9672 differentially methylated probes (DMPs) associated with DEX treatment during the first trimester of children at risk for, but not having, congenital adrenal hyperplasia [26]. For instance, they observed hypermethylation of specific CpG sites of *NR3C1*, *FKBP5,* and *SLC6A4* in DEX-treated individuals [26].

Following this line of research, the present study aimed to investigate whether DNA_M_ profiles and respective changes in the gene expression of stress-related candidate genes (*NR3C1, FKBP5, SLC6A4*) mediate the persistent effects of sGC on cortisol reactivity to a standardized laboratory stressor that have been observed previously by our group [4, 5] and others (e.g. Edelmann et al. [27]). For this, we collected blood samples for DNA_M_ and gene expression analyses from our children and adolescent cohort of mothers with a pathophysiological pregnancy (PP) who had received sGC treatment during pregnancy (PP/GC group) and controls from physiological pregnancies without complications. A second control group was further recruited to separate effects of sGC treatment from those related to maternal stress induced by the threat of preterm delivery and antepartum hospitalization. This group comprised children/adolescents of mothers who had been admitted to the hospital for serious pregnancy complications but had never received sGC therapy (PP/non-GC group). In contrast to most prior studies that included preterm infants with low birth weight (LBW), our group further aimed to disentangle the direct effects of antenatal sGC on DNA_M_ profiles from the confounding effects of preterm delivery [28] and LBW [29]. To this end, only term-born individuals with normal birth weight were enrolled in the current study. With regard to the assessment of HPA axis functioning, prior research in this field was mostly limited to acute markers reflecting short-term hormone levels. Addressing the gap, we aimed to determine both acute cortisol stress reactivity and hair cortisol concentrations (hairC), which have been proven a reliable and valid marker of long-term HPA-axis activity over a period of approximately three months [30]. Interestingly, these two markers are only poorly correlated and thus reflect different aspects of HPA-axis functioning [31, 32].

## Material and methods

### Participants

The actual sample consisted of two cohorts including a total of 57 term-born children (7–12 years) and 51 adolescents (14–18 years), both comprising PP/GC, PP/non-GC, and controls.

### Recruitment procedure

#### Children’s cohort

In cooperation with the Department of Gynecology and Obstetrics at the TU Dresden, medical reports from all mothers who delivered their babies between 2005 and 2010 were screened. From these 8421 mother/child dyads, only term-born offspring (≥37 weeks of gestation) who were not exposed to pediatric intensive care were considered as potential participants. Mothers were excluded from the study if the following exclusion criteria were fulfilled, i.e., serious diseases during pregnancy (metabolic diseases, gestational diabetes, placental insufficiency, preeclampsia, or known addiction), in order to avoid confounding effects of fetal lung maturation. Among these mothers, all who had been hospitalized for specific pregnancy complications, i.e., premature labor pain and/or vaginal bleeding and/or cervical insufficiency, were invited to take part in the study (*n* = 765). The remaining sample of hospitalized women consisted of two groups, namely mothers who received sGC therapy to accelerate fetal lung maturation (PP/GC, *n* = 523) and mothers who did not receive sGC treatment (PP/non-GC, *n* = 241). The PP/GC group was treated with either a single course of 12 mg of BETA administered twice over a 24 h interval or with DEX administered in four doses of 6 mg every 12 h during the 30^th^ week of gestation on average. Additionally, mothers with physiological pregnancies were invited as controls (*n* = 502). In total, 81 mother/child dyads participated in the main study [33]. From this sample, 57 mother/child dyads agreed to donate blood for DNA_M_ and mRNA expression analyses (which was an optional part of the main study), including 19 PP/GC, 13 PP/non-GC, and 25 controls.

#### Adolescent cohort

Medical reports from all mothers who delivered their babies between 1997 and 2003 were screened (for a detailed recruitment protocol, see [5]). Considering the above-mentioned inclusion and exclusion criteria, the PP/GC group included 304 mothers and the PP/non-GC group included 212 mothers (controls, *n* = 372). The PP/GC group was treated with the same BETA or DEX administration as described for the children’s cohort. In the first wave of testing (e.g., for the assessment of acute cortisol stress reactivity [4]), a total of 209 children took part in the study (PP/GC group, *n* = 81; PP/non-GC group, *n* = 43; controls, *n* = 85). In the actual second wave of testing, a subsample was re-invited in their adolescence for the analysis of acute cortisol stress reactivity [5], long-term hair steroids [33], and DNA methylation and mRNA expression of stress-associated genes (*NR3C1, FKBP5, SLC6A4*). Thus, all participants from the first study wave who (i) fulfilled inclusion criteria, and (ii) were willing to participate in the second study wave, and (iii) agreed to donate blood for DNA methylation and mRNA expression analysis (which was an optional part of the main study) were tested, including 23 PP/GC group, 7 PP/non-GC, and 21 controls.

### Study procedure

For the assessment of demographic characteristics (age, sex), birth-related characteristics (birth weight, birth length, APGAR score 5 minutes after birth, length of gestation), health-related variables (smoking, oral contraceptive, prenatal stress exposure), and hair related characteristics (number of hair washes per week, hair color), children/adolescents and their parents filled out a set of questionnaires. All participants gave written informed consent and the study was approved by the local ethic committee of the TU Dresden (EK 235062014) following the principles of the Declaration of Helsinki.

### Standardized laboratory stress test (TSST)

For stress induction, adolescents were exposed to the Trier Social Stress Test (TSST [34]), which is a standardized protocol that reliably elicits significant subjective and endocrine stress responses, including cortisol. Likewise, the children cohort underwent the Trier Social Stress Test for Children [35]. Detailed protocols have been published elsewhere [4, 5]. Saliva samples were collected 5 minutes before the TSST (baseline) as well as 10, 20, and 30 min after TSST onset. All test sessions were scheduled in the afternoon (1400–1800 h) to reduce variance in cortisol concentrations due to circadian secretion rhythms. Participants were asked to reschedule the session if feeling significantly impaired due to any reason and to refrain from physical exercising, smoking, eating, and drinking anything but water 1 h before the TSST.

### Saliva cortisol analysis

Saliva samples were collected with swabs (Cortisol Salivette®; Sarstedt, Nürmbrecht, Germany). Participants chew on the swabs for 30–60 s to stimulate saliva flow. Saliva samples were stored at −20 °C until the samples were thawed and centrifuged at 3000 rpm for 3 min. Salivary cortisol was analyzed using a commercial chemiluminescence immunoassay (CLIA; IBL, Hamburg, Germany) with intra-assay precision of 3.0% and inter-assay precision of 4.2%.

### Hair cortisol analysis

For the analyses of hair cortisol concentrations (hairC) scalp-near hair strands (~3 mm in diameter) were taken from a posterior vertex position. Hair strands were wrapped in aluminum foil and stored dry and dark until analysis. For the assessment of cumulated cortisol secretion over a 3-months period prior to sampling, hair segments of 3 cm were analyzed. HairC was analyzed by liquid chromatography coupled with tandem mass spectrometry (LC-MS/MS) as described in detail elsewhere [36].

### DNA methylation analysis

DNA from all participants was extracted from frozen EDTA-blood according to a simple salting out procedure [37] before 1 µg of DNA from each subject was bisulfite-treated using the EZ DNA Methylation-Gold^TM^ kit (Zymo, USA). DNA_M_ levels at specific genomic sites were investigated by targeted deep bisulfite sequencing as described elsewhere [38]. We focused on three regulatory regions of stress-associated candidate genes: 42 CpGs from *NR3C1*-1F promoter region (e.g. refs. [12, 39]), 84 CpGs from the *SLC6A4* 5′ regulatory region (e.g. refs. [19, 20]) and 5 CpGs located in intron 7 of *FKBP5* (e.g. Klengel et al. [14]) (see Supplementary Information 1 including Suppl. Fig. 1A–C and Suppl. Table 1A–C for exact chromosomal positions). The pair-end sequencing was performed on a MiSeq system (Illumina; San Diego – USA) using the Illumina MiSeq reagent Kit v2 (500 cycles- 2 × 250 paired end) in collaboration with the BioChip Labor of the Center of Medical Biotechnology (ZMB, University Essen/ Duisburg). A detailed laboratory protocol and primer sequences have been published elsewhere [38].

### RNA quantification by rt-qPCR

Blood was collected in PAXgene Blood RNA Tubes and total RNA extracted using the PAXgene Blood RNA Kit (PreAnalytiX GmbH, Switzerland). Subsequently, cDNA synthesis was conducted from 500 ng RNA using the iScript cDNA Synthesis Kit (Bio-Rad, USA) followed by real-time quantitative PCR analyses on a CFX384 realtime cycler (Bio-Rad, USA). A detailed protocol and primer sequences specially targeting mRNA of different housekeeping genes and three genes of interest (*NR3C1, FKBP5, SLC6A4)* are provided in Supplementary Information 2 and Suppl. Table 2. With respect to the retest reliability of mRNA expression profiles, a pilot study by our group already indicated a substantial trait component regarding *SLC6A4* mRNA expression [22]. Doing geNorm analysis [40], a set of nine candidate housekeeping genes in a representative set of 18 independent samples was assayed for stable mRNA expression. Result of this analysis was the selection of the most stably expressed set of three housekeeping genes: Beta-2-Microglobulin (*B2M*), Peptidylprolyl Isomerase A (*PPIA*), and Transferrin Receptor (*TFRC*) (Suppl. Fig. 2). Relative mRNA quantification analysis was performed using the qBase method [41]. All RT-qPCR analyses were performed in triplicates.

### Statistical analysis

Analyses were performed using R statistical programming (version 4.1). All statistical tests were two-tailed and a *P*-value of <0.05 was defined as statistically significant. Chi-squared tests for dichotomous and analyses of variance (ANOVAs) for continuous outcomes were used to examine group differences (PP/GC, PP/non-GC, controls) regarding demographic, as well as birth- and health-related characteristics. DNA_M_ at single CpG sites as well as mean DNA_M_ levels across the investigated genomic regions were analyzed. Given the large number of CpG sites quantified for *SLC6A4*, principal component analysis was conducted to reduce data into co-methylated factors (Supplementary Information 3 including Suppl. Fig. 3 and Suppl. Table 3). Tests for potential confounders regarding DNA_M_, mRNA expression, and cortisol values were assessed using correlations and ANOVAs (Supplementary Information 4 and Suppl. Table 4). Identified confounders, i.e., variables that were significantly associated with the exposure or the outcome, were included as covariates in the following analyses. In case of deviation from normal distribution (assessed with Kolmogorov–Smirnov test) of absolute DNA_M_, mRNA expression and/or cortisol values, subsequent analyses were based on natural log-transformed values. Non-parametrical statistical methods were used if log-transformation did not result in normality. In case of outliers, i.e., individuals who showed studentized residuals ≥ |3| regarding mean DNA_M_, mRNA expression and/or cortisol values, analyses will be run with and without outlier values to examine whether the outliers change the main findings. For descriptive purposes, mean data in figures were presented in original units.

First, we compared the mean DNA_M_ levels over all corresponding CpG sites per analyzed gene and mRNA expression levels between the three groups. The equality of variances was tested with Levene’s test. Next, separate ANOVAs or Kruskal–Wallis tests, respectively, were used to compare the groups. Second, we considered differences in DNA_M_ levels between the three groups at single CpG sites and co-methylated factors (in case of *SLC6A4*) by performing separate ANOVAs. All *p*-values were adjusted by Benjamini and Hochberg [42] correction for multiple testing by the number of CpG sites tested for each candidate gene (factors for *SLC6A4*). Further, linear regression analyses were conducted to test whether DNA_M_ predicted mRNA expression levels. In addition, covariate-adjusted regression models were performed to test whether (1) DNA_M_ (mean DNA_M_, at single CpG sites, co-methylated factors) and (2) mRNA expression were related to differences in cortisol stress reactivity (indexed by the cortisol area under the curve with respect to increase (AUCi) according to [43]) Further, covariate-adjusted regression models were set up to test whether (1) DNA_M_ (mean DNA_M_, at single CpG sites, co-methylated factors) and (2) mRNA expression was related to differences in hairC. In addition to frequentist analyses, Bayes factors were calculated for all tested hypotheses to examine the likelihood of the alternative (H1) compared to the null (H0) hypothesis [44]. Bayes factor above 1 is considered in favor of the H1 and a common convention is to interpret a Bayes factor ≥3 as moderate (and ≥10 as strong) evidence for the H1 [44, 45]. Contrarily, Bayes factor below 1 is considered as evidence for the H0, with values ≤1/3 indicating moderate (and ≤1/10 strong) evidence for the H0.

### Preregistration

This study was preregistered prior to analysis of the data (Open Science Framework; https://osf.io/akdtv/).

## Results

### Sample characteristics

The present study cohort included 108 healthy, term-born children and adolescents (42 PP/GC, 20 PP/non-GC, 46 controls). Group differences regarding demographic, birth- and health-related characteristics are shown in Table 1. The groups did not significantly differ with respect to sex (*p* = 0.349), age (*p* = 0.649) or BMI (*p* = 0.366). Although all participants were term-born (>37 weeks of gestation), significant group differences were observed in length of gestation (*p* = 0.038), showing that individuals in the PP/GC group were born earlier than individuals in the comparison groups. The groups did not differ in terms of other birth-related characteristics (birth weight, birth length, APGAR index at 5 min after birth; all *p* ≥ 0.190), or prenatal stress exposure (*p* = 0.673). Thus, length of gestation was included as a covariate into the following analyses testing for group differences. In previous studies, we had already shown that the PP/GC group was characterized by higher cortisol levels in response to the TSST both during childhood [4] and late adolescence [5] as compared to controls, whereas no group differences were found with regard to hairC [33].

**Table 1:** Sample characteristics (*N* = 108).

	PP/GC	PP/non-GC	Controls	*P value*
*N*	42	20	46	
Age (y)	12.60 ± 4.32	11.60 ± 3.35	12.15 ± 3.92	0.649
Sex (% male)	69.05	50.00	63.04	0.349
BMI	18.85 ± 3.26	17.46 ± 4.22	18.70 ± 3.23	0.366
Birth-related characteristics				
Birth weight (g)	3217.82 ± 510.03	3239.00 ± 533.90	3300.12 ± 510.61	0.768
Birth length (cm)	50.49 ± 2.25	49.10 ± 2.92	50.15 ± 2.50	0.190
APGAR 5 min	9.18 ± 0.79	8.95 ± 0.76	9.25 ± 0.54	0.322
Length of gestation	38.78 ± 1.41	39.62 ± 0.85	39.42 ± 1.33	0.038
Health-related characteristics				
Prenatal stress exposure	2.86 ± 2.16	2.55 ± 1.57	2.52 ± 1.67	0.673

Mean ± SD.

*BMI* body mass index, *PP* pathophysiological pregnancy, *GC* glucocorticoid.

### DNA methylation analysis

On average, assays showed around 8247.26 (±5777.06) reads per sample and gene (Max = 28814, Min = 467). The DNA_M_ analysis resulted in a mean methylation value of 91.36% (SD = 1.53%) for *FKBP5* and 5.12% (SD = 0.65%) for *SLC6A4*. As also observed by others (e.g. refs. [38, 39, 46]), the analyzed *NR3C1* stretch was generally unmethylated, with mean DNA_M_ values below 1% (Suppl. Fig. 1A). Given the low mean DNA_M_ level (*M* = 0.66%) as well as the low variability (SD = 0.10%) of *NR3C1*, no further statistical analyses were performed. For *FKBP5* and *SLC6A4*, no association of DNA_M_ with mRNA expression could be observed (for details see Supplementary information 5 and Suppl. Fig. 4).

### No effects of antenatal sGC treatment on DNA_M_ or mRNA expression in stress-associated genes

**Fig. 1:**
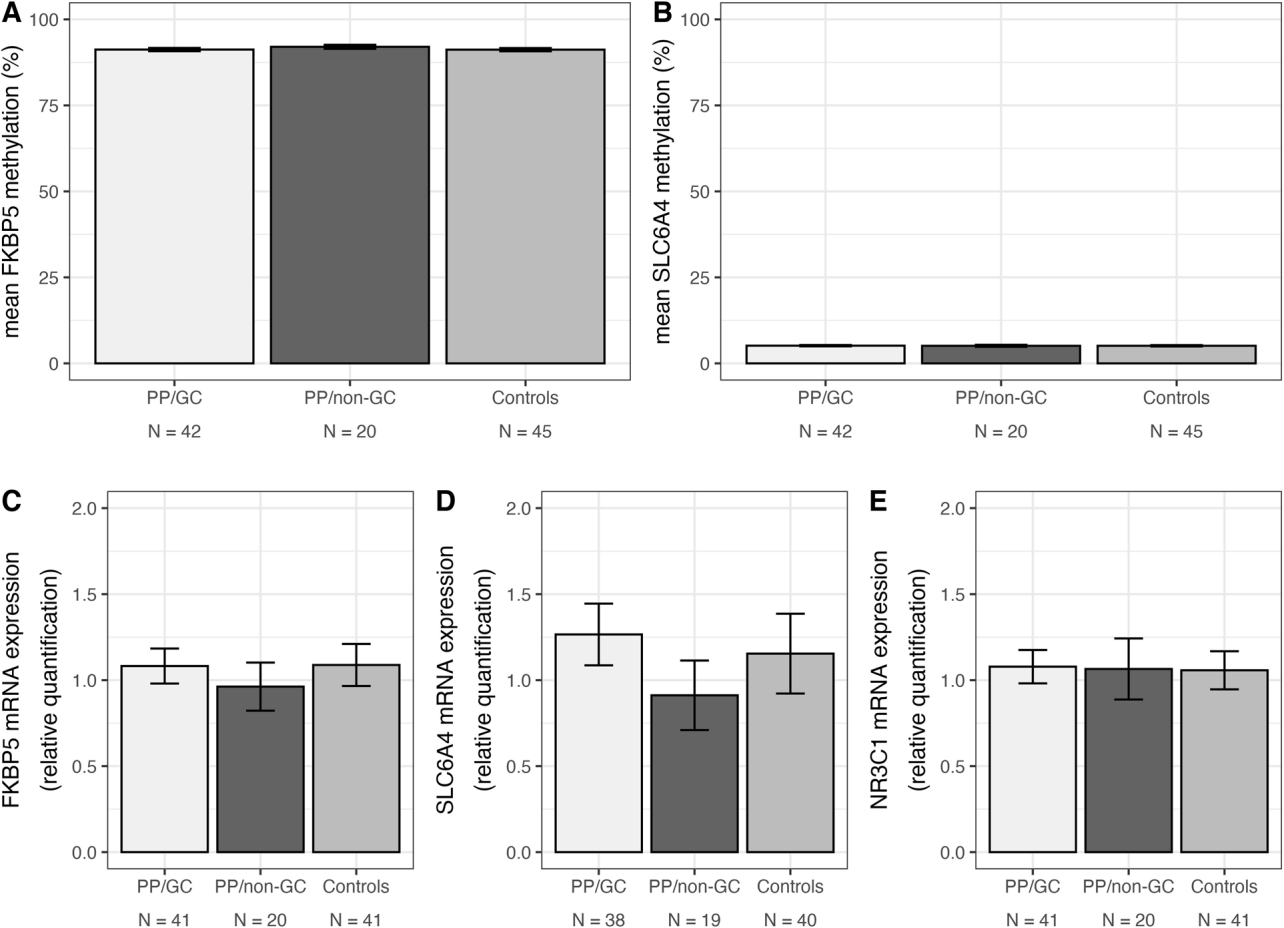
Effects of antenatal sGC treatment on DNA methylation and mRNA expression. Upper part: There were no significant differences in mean DNA methylation levels between the three groups (pathological pregnancy group with antenatal synthetic glucocorticoid treatment (PP/GC), pathological pregnancy group without antenatal synthetic glucocorticoid treatment (PP/non-GC), untreated control group from physiological pregnancies). Data show mean with 95% confidence interval. **A** Mean DNA methylation of *FKBP5*. **B** Mean DNA methylation of *SLC6A4*. Lower part: There were no significant differences in mRNA expression levels between the three groups. **C** mRNA expression of *FKBP5*. **D** mRNA expression of *SLC6A4*. **E** mRNA expression of *NR3C1*.

First, group differences (PP/GC, PP/non-GC, controls) in mean DNA_M_ (*FKBP5*, *SLC6A4*) and mRNA expression levels (*NR3C1*, *FKBP5*, *SLC6A4*) were analyzed by means of separate ANOVAs or Kruskal-Wallis-tests, respectively. In the overall sample, no significant differences in DNA_M_ between the three groups in any of the genes (*FKBP5*: *χ*²(2) = 5.61, *p* = 0.061; BF_10_ = 0.574; *SLC6A4*: *F*(2,104) = 0.05, *p* = 0.955; BF_10_ = 0.096; Fig. 1A, B) were observed. Likewise, no significant differences in mRNA expression between the three groups in any of the genes (*NR3C1*: χ²(2) = 0.33, *p* = 0.846; BF_10_ = 0.098; *FKBP5*: *F*(2,100) = 1.00, *p* = 0.370; BF_10_ = 0.202; *SLC6A4*: *F*(2,95) = 2.75, *p* = 0.069; BF_10_ = 0.845; Fig. 1C–E) were observed. For completeness, we also tested for group differences in DNA_M_ at specific CpG sites (*FKBP5*, *SLC6A4*) and co-methylated factors (*SLC6A4*) by replacing mean DNA_M_ by DNA_M_ at five individual CpG sites for *FKBP5*, 84 individual CpG sites and two co-methylated factors for *SLC6A4* in the ANOVA. However, the results were largely comparable and showed no significant differences between the three groups for site- as well as factor-specific DNA_M_ after correction for multiple testing (number of CpGs tested per gene; all *P*_adj_ ≥ 0.190). Together, these findings indicate comparable DNA_M_ and mRNA expression levels in stress-associated genes in individuals of the PP/GC, PP/non-GC, and control group. Results of the Bayes factor analyses largely confirmed the findings of the frequentist analyses. There was anecdotal to strong evidence for the H0 of no group differences of DNA_M_ and mRNA expression levels (for the results of Bayes factors analyses for single and site-specific DNA_M_ see Supplementary Information 6). Comparable results were achieved when controlling for potential confounding effects and after exclusion of identified outliers.

### No associations of DNA_M_ and mRNA expression with cortisol stress reactivity

**Fig. 2:**
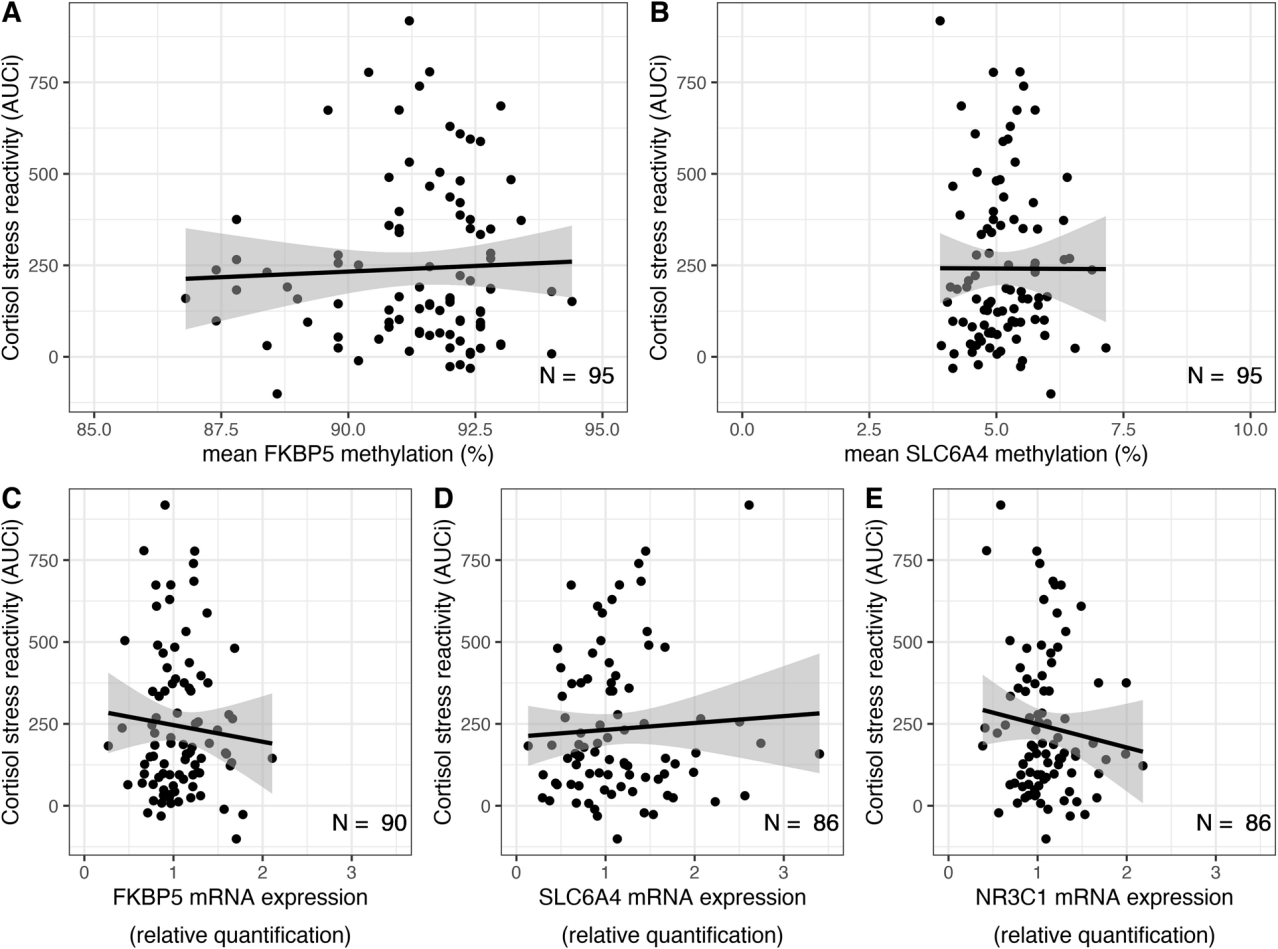
Association of DNA methylation and mRNA expression with cortisol stress reactivity. Upper part: Mean DNA methylation levels were not significantly related to cortisol stress reactivity (indexed by the cortisol AUCi). **A** Mean DNA methylation of FKBP5. **B** Mean DNA methylation of SLC6A4. Lower part: mRNA expression levels were not significantly related to cortisol stress reactivity. **C** mRNA expression of FKBP5. **D** mRNA expression of SLC6A4. **E** mRNA expression of NR3C1.

Next, we investigated whether (1) DNA_M_ and (2) mRNA expression were related to differences in cortisol stress reactivity. We set up regression models to test whether (1) mean DNA_M_ and (2) mRNA expression were related to differences in AUCi cortisol stress reactivity. In resulting models, neither mean DNA_M_ (*FKBP5*: *t*(93) = 0.43, *p* = 0.672, *β* = 0.04; BF_10_ = 0.234; *SLC6A4*: *t*(93) = –0.02, *p* = 0.983, *β* = 0.002; BF_10_ = 0.216; Fig. 2A, B) nor mRNA expression (*NR3C1*: *t*(88) = –1.02, *p* = 0.309, *β* = –0.11; BF_10_ = 0.349; *FKBP5*: *t*(88) = –0.72, *p* = 0.476, *β* = –0.08; BF_10_ = 0.276; *SLC6A4*: *t*(84) = 0.54, *p* = 0.594, *β* = .06; BF_10_ = 0.255; Fig. 2C–E) were significantly related to cortisol stress reactivity. Again, we also tested whether DNA_M_ at specific CpG sites (*FKBP5*, *SLC6A4*) and of co-methylated factors (*SLC6A4*) was associated with cortisol release. However, the results were largely comparable and site- as well as factor-specific DNA_M_ was unrelated to cortisol stress reactivity (all *P*_adj_ ≥ 0.202). Together these findings do not provide evidence for an association of DNA_M_ and mRNA expression with cortisol stress reactivity. The Bayes factor analyses suggested that there is anecdotal to moderate evidence for the H0 of no effects of DNA_M_ and mRNA expression on cortisol stress reactivity. Comparable results were achieved when controlling for potential confounders.

### No associations of DNA_M_ and mRNA expression with hairC

**Fig. 3:**
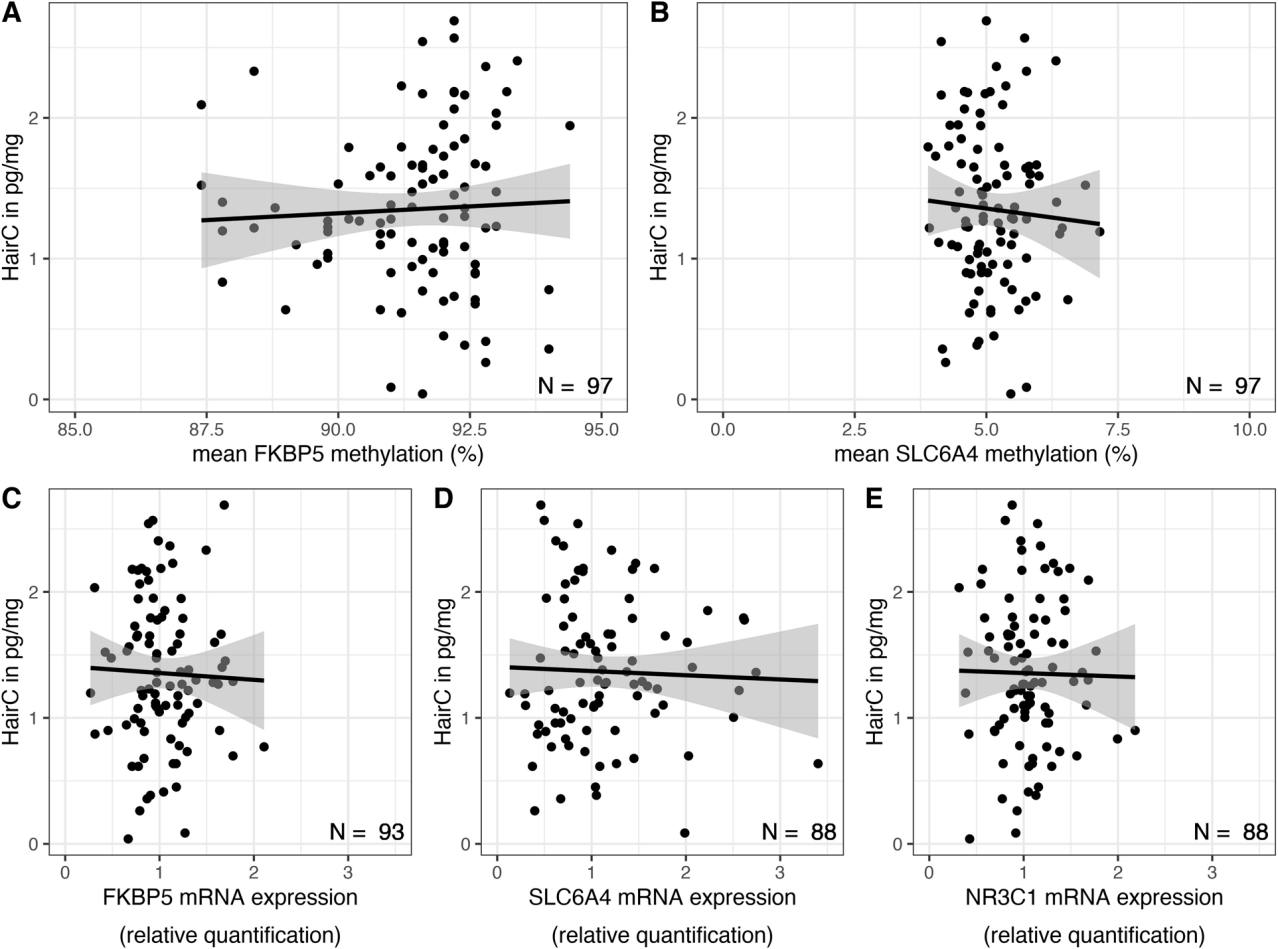
Association of DNA methylation and mRNA expression with hairC. Upper part: Mean DNA methylation levels were not significantly related to hair cortisol concentration (hairC). **A** Mean DNA methylation of *FKBP5*. **B** Mean DNA methylation of *SLC6A4*. Lower part: mRNA expression levels were not significantly related to hairC. **C** mRNA expression of *FKBP5*. **D** mRNA expression of *SLC6A4*. **E** mRNA expression of *NR3C1*.

Next, we set up regression models to test whether (1) mean DNA_M_ and (2) mRNA expression were related to differences in hairC. In resulting models, neither mean DNA_M_ (*FKBP5*: *t*(95) = 0.48, *p* = 0.630, *β* = 0.05; BF_10_ = 0.237; *SLC6A4*: *t*(95) = –0.56, *p* = 0.575, *β* = –0.06; BF_10_ = 0.246; Fig. 3A, B) nor mRNA expression (*NR3C1*: *t*(91) = –0.15, *p* = 0.880, *β* = –0.02; BF_10_ = 0.220; *FKBP5*: *t*(91) = –0.31, *p* = 0.757, *β* = –0.03; BF_10_ = 0.220; *SLC6A4*: *t*(86) = –0.34, *p* = 0.734, *β* = –0.04; BF_10_ = 0.235; Fig. 3C–E) were significantly related to hairC. Again, we also tested whether DNA_M_ at specific CpG sites (*FKBP5, SLC6A4*) and co-methylated factors (*SLC6A4*) was associated with hairC. However, the results were largely comparable and site- as well as factor-specific DNA_M_ was not significantly related to hairC (all *P*_adj_ ≥ 0.845). Together these findings do not provide evidence for an association of DNA_M_ and mRNA expression with hairC. The Bayes factor analyses provided moderate evidence for the H0 of no effects of DNA_M_ and mRNA expression on HairC. Comparable results were achieved when controlling for potential confounders.

## Discussion

The present study investigated whether DNA_M_ and respective mRNA expression profiles of three stress-related candidate genes (*NR3C1, FKBP5, SLC6A4*) act as mediators of the persistent effects of sGC treatment on cortisol reactivity that have been previously observed by our group [4, 5]. For this, DNA_M_ and mRNA expression profiles of children/adolescents from PP who had received sGC treatment during pregnancy (DEX or BETA) were compared to controls from pregnancies without complications. In order to separate the direct effects of sGC exposure from those related to maternal stress induced by antenatal hospitalization, a second control group was recruited consisting of children/adolescents of mothers who had been admitted to hospital for serious pregnancy complications but did not receive any sGC therapy. Importantly, the current sample included only term-born individuals to exclude the confounding effects of preterm delivery [28] and LBW [29] on DNA_M_ profiles. We found no evidence of group differences in DNA_M_ and mRNA expression levels of three stress-associated genes (*NR3C1, FKBP5, SLC6A4)*. Likewise, no associations of respective DNA_M_/mRNA expression levels with acute cortisol stress reactivity or long-term cortisol levels in hair were observed.

While the fetal epigenome is particularly responsive to intrauterine stress exposures [8], recent efforts to generate robust associations with DNA_M_ at specific stress-related candidate loci remained challenging. Expanding prominent findings from rodent models [12], a number of human studies tried to provide evidence for a link between childhood (e.g. van der Knaap et al. [47]) and in utero (for a meta-analysis see [11]) adversity and increased *NR3C1-1F* DNA_M_, rendering this locus a potential candidate for investigating effects of sGC treatment. However, an increasing number of studies could not demonstrate such an effect (e.g. refs. [48, 49]), and one important message from human studies targeting *NR3C1-1F* was that overall DNA_M_ levels measured in peripheral and hippocampus cells were generally low with limited sample variance observed for most individual sites [39, 46, 48]. In the present study, the *NR3C1*-1F promoter region was also found to be largely unmethylated and thus was excluded from further analyses (Suppl. Figure 1A). Although subtle differences in DNA_M_ may theoretically promote changes in HPA-axis functioning, it is important to note that even the most sensitive method like targeted deep bisulfite sequencing is not able to reliably detect DNA_M_ below 1% [38]. Beyond the question of their biological plausibility [50], these technical restrictions should be carefully considered when interpreting previously observed effects of early environmental adversity on *NR3C1-*1F DNA_M_ levels.

In addition to *NR3C1*-1F, DNA_M_ changes within the *FKBP5* gene were considered as a second promising mediator of altered HPA-axis functioning following antenatal sGC treatment. Several previous studies reported demethylation of a GRE located in intron 7 of the *FKBP5* gene following early environmental adversity (e.g. Klengel et al. [14]), but this could not be confirmed by others (e.g. Alexander et al. [15]). In contrast, different forms of maternal stress (e.g., maternal affective disorders and perceived distress) were repeatedly associated with increased *FKBP5* DNA_M_ in cord blood and placental samples, although these studies targeted different regions of the *FKBP5* gene [17, 18]. Of particular relevance, in vitro dexamethasone treatment has been found to induce an active demethylation of *FKBP5* intron 7 in human hippocampal progenitor cell lines, which may impose an increased risk for a chronic state of hypercortisolism [14]. While we observed substantial DNA_M_ variability in our sample, our findings revealed no effects of sGC treatment on *FKBP5* DNA_M_ and respective mRNA expression levels. In accordance with an independent study on healthy adults by our group [15], our data provide no evidence for a biological relevance of *FKBP5* DNA_M_ levels in terms of changes in mRNA expression or acute and chronic cortisol output. The latter findings thus conflict with studies reporting negative associations of *FKBP5* intron 7 DNA_M_ and single measures of early morning cortisol concentrations [51] as well as averaged cortisol awakening levels sampled over one month [52].

Lastly, we targeted a promoter-associated CpG island in the *SLC6A4* gene that has already been linked to early environmental adversity and HPA-axis alterations in previous research (for a review see [9]). The few studies that investigated prenatal stress exposures produced overall conflicting results, including a negative correlation between *SLC6A4* DNA_M_ levels and maternal depressive mood during pregnancy [21] as well as no association with different maternal prenatal stressors (e.g. Wankerl et al. [22]). In turn, site-specific changes in *SLC6A4* DNA_M_ levels have been linked to increased cortisol stress reactivity in studies depending on an individual’s genetic predisposition [19, 53], while this link was not observed by others [54]. Findings of the current study, however, could not provide further evidence that DNA_M_ and respective mRNA expression profiles of *SLC6A4* are sensitive to prenatal sGC treatment or play a key mediating role in its persisting effects on HPA-axis functioning.

Several limitations of the present study have to be considered. First, the number of mother/child dyads who agreed to donate blood was relatively small. Second, we did not account for genetic variants that are known to moderate associations of early adversity on DNA_M_ profiles [14] and may also amplify epigenetic effects on HPA-axis functioning [19, 20, 55]. The presence of genetic variants throughout the whole genome affecting DNA_M_ pattern cannot be excluded [56]. Particularly with regard to hairC, which is a highly heritable marker [57], the interplay between genetic and epigenetic effects should be further elucidated in future studies [55]. Third, DNA_M_ of candidate genes was determined in surrogate peripheral tissue. Even though a genome-wide study showed significant correlations between DNA_M_ in the live human brain and whole blood within individuals (for brain-blood correlation see website IMAGE-CpG [58]: *FKBP5*: *r* = 0.95; *SLC6A4*: *r* = 0.94), the relevance for crucial brain structures involved in HPA-axis regulation (e.g. the hippocampus) is not yet reliably proven. Fourth, the mixture of cell types in whole blood samples introduces a potential bias due to possible significant differences in both the level and variability of DNA_M_ between different sample types (e.g. whole blood vs. homogeneous cell types) [59]. In accordance with previous studies, cell type heterogeneity may constitute a potential confounder of DNA_M_ profiles (e.g. Jones et al. [60]).

In summary, our study provides no evidence for a substantial effect of prenatal sGC treatment on *NR3C1, FKBP5,* and *SLC6A4* DNA_M_ levels in late childhood or adolescence that compares to those observed in the aftermath of prenatal stress in other studies (e.g. *NR3C1* [11]; *FKBP5* [17]; *SLC6A4* [21]). Although cortisol excess reflects a core mediator of maternal stress transfer to the fetal epigenome [24], it is important to note that the effects of maternal GC may differ from those of sCG used in neonatal practice (e.g. sGCs readily cross the placenta whereas maternal GCs are converted into inactive cortisone [61]). Moreover, inconsistent findings might result from differences regarding the choice of the specific tissue used to investigate epigenetic markers as well as different analytical methods used to quantify DNA_M_. Consequently, the mechanisms underlying the persistent effects of sGC on HPA-axis functioning observed in our sample remain unknown. Future studies should consider other mechanisms to examine these effects on stress reactivity, e.g. by including functional measures of GR sensitivity [49]. Future genome-wide methylation analysis studies might shed light on potentially unexplored epigenetic mediators in this context, however, they would require very large samples to achieve adequate statistical power. A first small-scaled epigenome-wide association study on 29 children at risk for, but not having, congenital adrenal hyperplasia already provided preliminary evidence for 9672 DMPs associated with DEX treatment during the first trimester of pregnancy [26]. Functional enrichment of those DMPs was mainly associated with immune functioning and inflammation, however, a set of DMPs was also implicated in steroidogenesis, thus highlighting the potential relevance for long-term cortisol output. Regarding the use of genome-wide DNA_M_ data, epigenetic scores (ES) might reflect a powerful strategy to aggregate effects of single loci, which can then serve to robustly predict changes in central response systems even in smaller samples. In a landmark study of human HPCs, sGC were recently found to induce widespread changes of DNA_M_ at sites involved in cellular and organ development, transcription, neurogenesis, and neuronal differentiation [25]. To account for the cross-tissue relevance of those GC-induced DNA_M_ changes, the authors calculated a weighted ES of those differentially methylated sites that were found to overlap in HPCs and peripheral human blood cells. Intriguingly, when applying this DEX-sensitive ES to DNA_M_ data of human newborn cord blood samples, it successfully predicted prenatal stress exposure and might thus offer an innovative future strategy to investigate molecular changes following antenatal sGC treatment.

## Supplementary information

Supplementary Material
