# Supplementary material for: No long-term effects of antenatal synthetic glucocorticoid exposure on epigenetic regulation of stress-related genes
Source: Transl Psychiatry. 2022 Feb 16;12:62. doi: 10.1038/s41398-022-01828-x (PMC8850596; doi:10.1038/s41398-022-01828-x)
Supplement: Supplementary file 1 — Supplementary Material [file 41398_2022_1828_MOESM1_ESM.docx]

**No Long-Term Effects of Antenatal Synthetic Glucocorticoid Exposure on Epigenetic Regulation of Stress-Related Genes**

**Müller, S.; Moser, D.; Frach, L.; Wimberger, P.; Nitzsche, K.; Li, S.-C.; Kirschbaum, C. & Alexander, N.**

**Supplementary Material**

**Supplement 1: DNA methylation analysis**

**
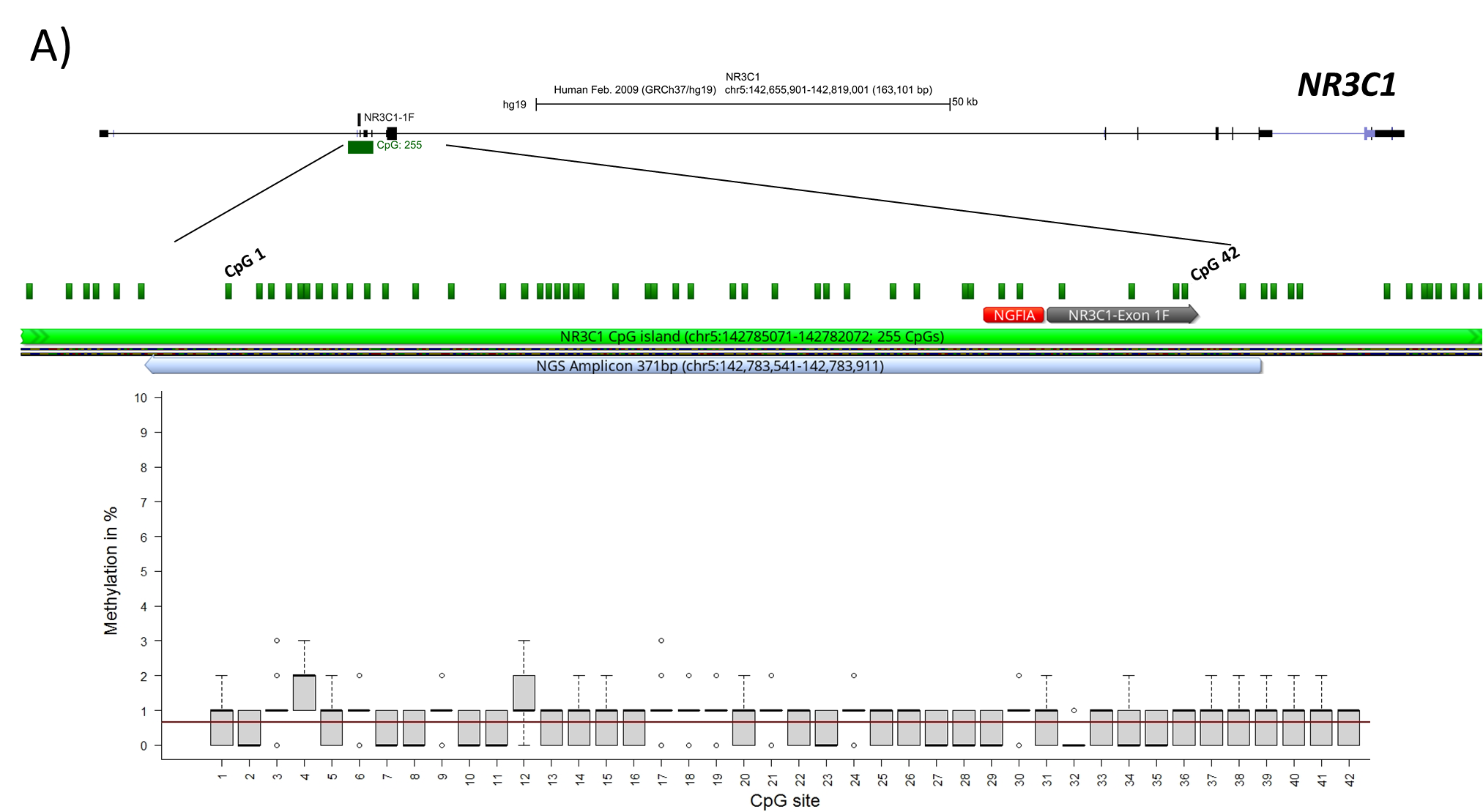
**

**
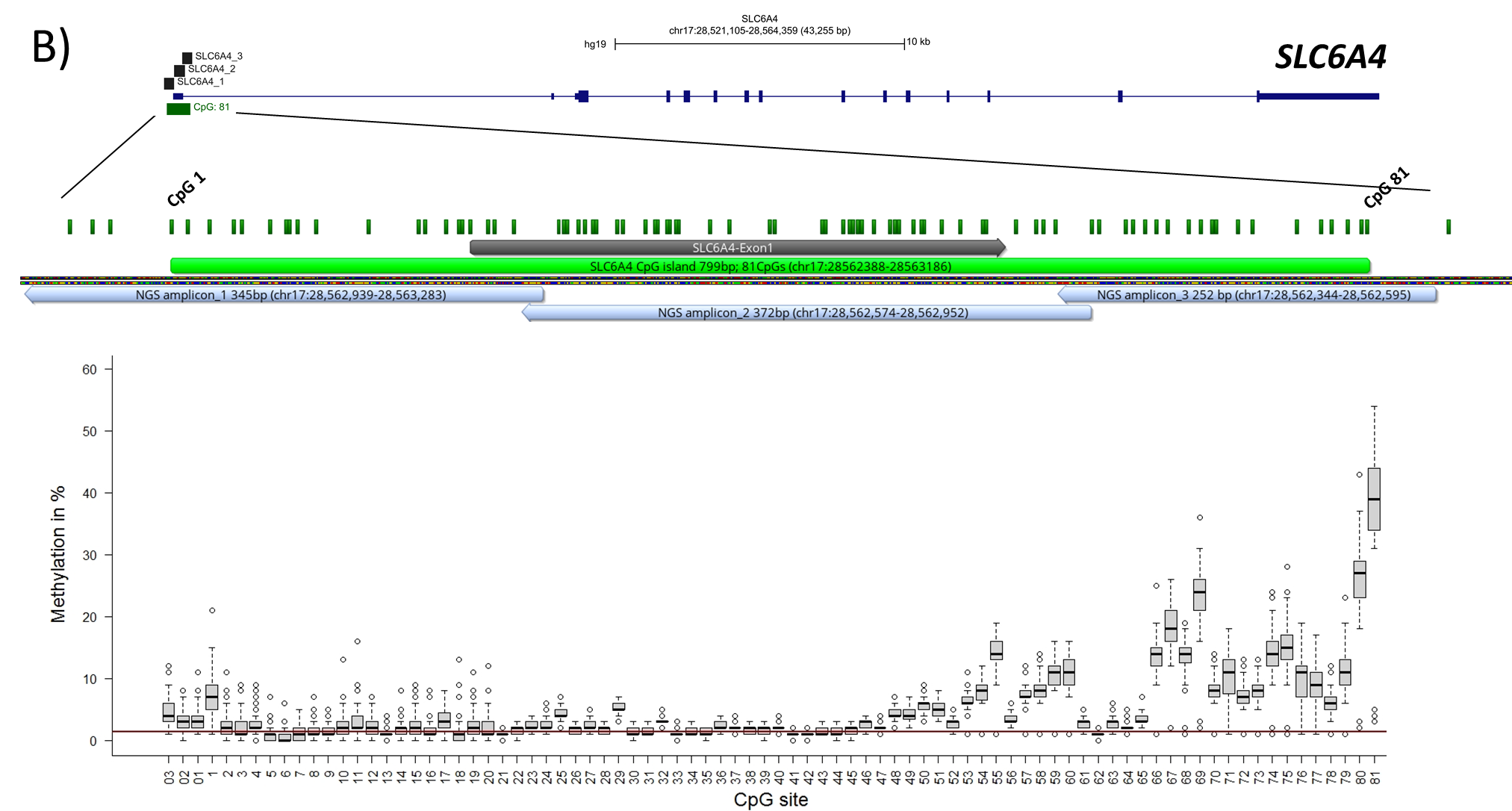
**

**
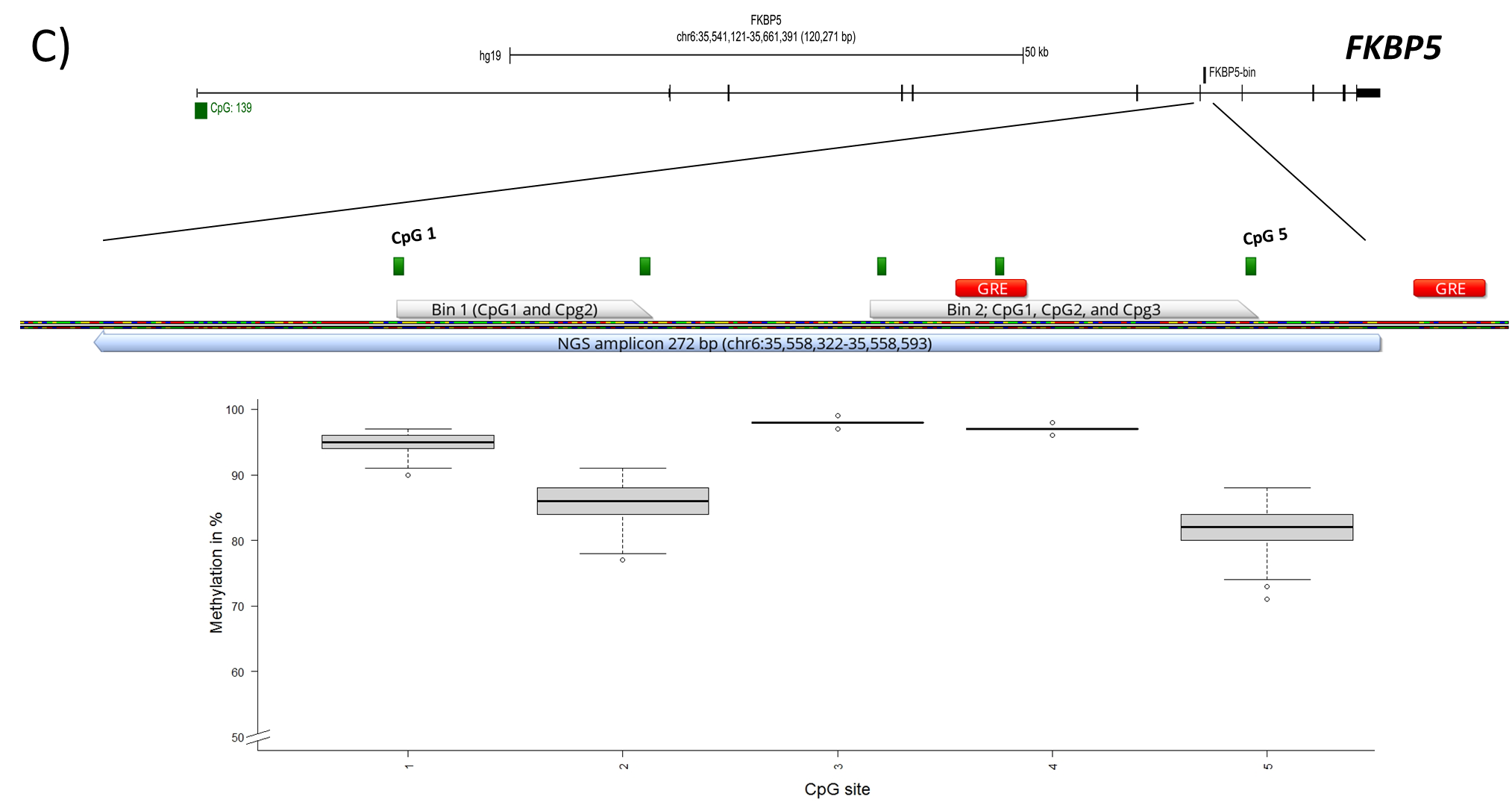
**

**Supplement Figure 1A-C: Chromosomal position of all genes and corresponding DNA methylation levels.**
Chromosomal position of all genes are illustrated using graphical outputs generated by the UCSC Genome Browser (https://genome.ucsc.edu) and Geneious Prime 2019 software (https://www.geneious.com). All genes are presented in 5’ -> 3’ orientation from left to right. Amplicons are highlighted with respect to their genomic orientation. All CpG sites are illustrated with green bars, CpG islands in light green, and exons in grey colour. Boxplots showing DNA methylation across the investigated CpG sites. The box covers the methylation data of each CpG site between the 25th to 75th quantile, the whiskers show the range of values failing within 1.5-fold the interquartile range. The horizontal line represents the methylation detection limit for targeted deep bisulfite sequencing (*NR3C1*: 0.67%; *SLC6A4*: 1.44%; *FKBP5*: 1.24%).

**Supplement table 1A-C: Chromosomal positions of CpG sites investigated for all genes.** Genomic sequence in 5 🡪 3 orientation of the gene fragments analysed. All CpG sites that were quantified for methylation are numbered in ascending order. Specific CpG sites included in epigenome-wide arrays with code numbers for the Infinium HumanMethylation450 BeadChip Kit (Illumina) and EPIC BeadChip Kit (Illumina).

**A) *NR3C1* (chr5:142,783,541-142,783,911)**

| **CpG number** | **chr5 position** | **Infinium 450K** | **Infinium EPIC** |
| --- | --- | --- | --- |
| CpG 1 | 142783883 |  |  |
| CpG 2 | 142783873 |  |  |
| CpG 3 | 142783869 |  |  |
| CpG 4 | 142783863 |  |  |
| CpG 5 | 142783859 |  |  |
| CpG 6 | 142783857 |  |  |
| CpG 7 | 142783853 |  |  |
| CpG 8 | 142783848 |  | cg21209684 |
| CpG 9 | 142783843 | cg18068240 | cg18068240 |
| CpG 10 | 142783837 |  |  |
| CpG 11 | 142783831 |  | cg14939152 |
| CpG 12 | 142783821 |  |  |
| CpG 13 | 142783809 |  |  |
| CpG 14 | 142783792 |  |  |
| CpG 15 | 142783785 |  |  |
| CpG 16 | 142783780 |  |  |
| CpG 17 | 142783777 |  |  |
| CpG 18 | 142783774 |  |  |
| CpG 19 | 142783771 |  |  |
| CpG 20 | 142783768 |  |  |
| CpG 21 | 142783766 |  |  |
| CpG 22 | 142783755 |  |  |
| CpG 23 | 142783744 |  |  |
| CpG 24 | 142783742 |  |  |
| CpG 25 | 142783735 |  |  |
| CpG 26 | 142783730 |  |  |
| CpG 27 | 142783716 |  |  |
| CpG 28 | 142783712 |  |  |
| CpG 29 | 142783702 |  |  |
| CpG 30 | 142783688 |  |  |
| CpG 31 | 142783685 |  |  |
| CpG 32 | 142783678 |  |  |
| CpG 33 | 142783663 |  |  |
| CpG 34 | 142783655 |  |  |
| CpG 35 | 142783639 | cg15645634 | cg15645634 |
| CpG 36 | 142783637 |  |  |
| CpG 37 NGFIA | 142783627 |  |  |
| CpG 38 NGFIA | 142783621 | cg15910486 | cg15910486 |
| CpG 39 | 142783607 | cg04111177 | cg04111177 |
| CpG 40 | 142783584 |  |  |
| CpG 41 | 142783569 | cg17860381 | cg17860381 |
| CpG 42 | 142783566 |  |  |

Note: Numbering of CpG 1 through 38 is identical to (1, 2)

**B) *SLC6A4* (chr17:28,562,344-28,563,283)**

| **CpG number** | **chr17 position** | **Infinium 450K** | **Infinium EPIC** |
| --- | --- | --- | --- |
| CpG 03 | 28563253 |  |  |
| CpG 02 | 28563237 |  |  |
| CpG 01 | 28563226 |  |  |
| CpG 1 | 28563185 |  |  |
| CpG 2 | 28563174 |  |  |
| CpG 3 | 28563159 |  |  |
| CpG 4 | 28563143 |  |  |
| CpG 5 | 28563138 |  |  |
| CpG 6 | 28563119 | cg27569822 |  |
| CpG 7 | 28563108 | cg10901968 | cg10901968 |
| CpG 8 | 28563106 |  |  |
| CpG 9 | 28563101 |  |  |
| CpG 10 | 28563089 | cg26741280 | cg26741280 |
| CpG 11 | 28563054 | cg25725890 | cg25725890 |
| CpG 12 | 28563020 |  |  |
| CpG 13 | 28563016 |  |  |
| CpG 14 | 28563002 |  |  |
| CpG 15 | 28562993 |  |  |
| CpG 16 | 28562991 |  |  |
| CpG 17 | 28562986 |  |  |
| CpG 18 | 28562974 |  |  |
| CpG 19 | 28562970 |  |  |
| CpG 20 | 28562957 |  |  |
| CpG 21 | 28562927 |  |  |
| CpG 22 | 28562923 |  |  |
| CpG 23 | 28562921 |  |  |
| CpG 24 | 28562914 |  |  |
| CpG 25 | 28562909 |  |  |
| CpG 26 | 28562904 |  |  |
| CpG 27 | 28562902 |  |  |
| CpG 28 | 28562888 |  |  |
| CpG 29 | 28562884 |  |  |
| CpG 30 | 28562869 |  |  |
| CpG 31 | 28562863 |  |  |
| CpG 32 | 28562861 |  |  |
| CpG 33 | 28562855 |  |  |
| CpG 34 | 28562853 |  |  |
| CpG 35 | 28562849 |  |  |
| CpG 36 | 28562847 |  |  |
| CpG 37 | 28562826 |  |  |
| CpG 38 | 28562813 | cg05016953 | cg05016953 |
| CpG 39 | 28562786 |  |  |
| CpG 40 | 28562783 |  |  |
| CpG 41 | 28562751 |  | cg06373684 |
| CpG 42 | 28562749 |  |  |
| CpG 43 | 28562737 |  |  |
| CpG 44 | 28562733 |  | cg26438554 |
| CpG 45 | 28562731 |  |  |
| CpG 46 | 28562728 |  |  |
| CpG 47 | 28562725 |  |  |
| CpG 48 | 28562717 |  |  |
| CpG 49 | 28562706 |  |  |
| CpG 50 | 28562703 |  |  |
| CpG 51 | 28562700 |  |  |
| CpG 52 | 28562691 |  |  |
| CpG 53 | 28562685 | cg14692377 | cg14692377 |
| CpG 54 | 28562683 |  |  |
| CpG 55 | 28562672 |  |  |
| CpG 56 | 28562659 |  |  |
| CpG 57 | 28562644 |  |  |
| CpG 58 | 28562642 |  |  |
| CpG 59 | 28562622 |  |  |
| CpG 60 | 28562609 |  |  |
| CpG 61 | 28562604 |  |  |
| CpG 62 | 28562596 |  |  |
| CpG 63 | 28562572 |  |  |
| CpG 64 | 28562567 |  |  |
| CpG 65 | 28562549 |  |  |
| CpG 66 | 28562544 |  |  |
| CpG 67 | 28562536 |  |  |
| CpG 68 | 28562529 |  |  |
| CpG 69 | 28562521 |  |  |
| CpG 70 | 28562507 |  |  |
| CpG 71 | 28562499 |  |  |
| CpG 72 | 28562492 |  |  |
| CpG 73 | 28562489 |  |  |
| CpG 74 | 28562474 | cg03363743 | cg03363743 |
| CpG 75 | 28562465 |  |  |
| CpG 76 | 28562435 |  |  |
| CpG 77 | 28562419 |  |  |
| CpG 78 | 28562412 |  |  |
| CpG 79 | 28562401 |  |  |
| CpG 80 | 28562392 |  |  |
| CpG 81 | 28562388 |  |  |

Note: Numbering of CpG 1 through 81 (799 bp CpG
island) is identical to (3)

**C) *FKBP5* (chr6:35,558,322-35,558,593)**

| **CpG number** | **CpG according to (4)** | **chr6 position** | **Infinium EPIC** |
| --- | --- | --- | --- |
| CpG 1 | Bin 1 - CpG1 | 35558386 |  |
| CpG 2 | Bin 1 - CpG2 | 35558438 |  |
| CpG 3 | Bin 2 - CpG1 | 35558488 | cg22363520 |
| CpG 4 | Bin 2 - CpG2 | 35558513 |  |
| CpG 5 | Bin 2 - CpG3 | 35558566 |  |

Note: No binding site on the Infinium 450K BeadChip

**Supplement 2: Quantitative RT-qPCR protocol**

RNA concentrations were quantified and RNA integrities analyzed using a Fragment Analyzer (Advanced Analytical, Agilent, USA). RNA samples had an average relative quality number (RQN) of 8.7 (*SD* = 0.48; Maximum = 10); three samples were excluded due to poor quality. Standard curves for each gene of interest and housekeeping gene were assayed in order to estimate PCR efficiency. Rt-qPCR experiments were conducted in a final volume of 10 µl containing 2 µl cDNA, 0.3 µl forward and reverse primers (10 µM) and 5 µl 2x SYBR-green MasterMix (iTaq, Biorad). Because of its low expression in blood cells, the linear range of *SLC6A4* detection was experimentally validated by limit of detection/ quantification (LOD/ LOQ) analysis as described elsewhere (5). As a result of the LOD/ LOQ, Rt-qPCR experiment of *SLC6A4* was conducted in a final volume of 12 µl containing 5 µl cDNA, 0.36 µl forward and reverse primers (10 µM) and 6 µl 2x SYBR-green MasterMix (iTaq, Biorad). The thermocycler protocol involved an initial denaturation (3 min, 95°C), 40 cycles of denaturation (0:05 min, 95°C), annealing (0:20 min, 60°C) and extension followed by a final melting curve analysis. Quality control including the geNorm expression stability value of the three housekeeping genes (*M* = 0.54) and the coefficient of variation of the normalized housekeeping genes relative quantities (*CV* = 0.22) was closed to the default settings for homogeneous samples set (*M* = 0.5, *CV* = 0.2; 6).

**Supplement table 2: Primer sequences for mRNA expression analysis.**Exon spanning Primers were designed using primer 3 software (<http://bioinfo.ut.ee/primer3-0.4.0/>).

| **Gene** | **Gene name** | **Primer** | **Sequence** | **Length Target [bp]** |
| --- | --- | --- | --- | --- |
| **Genes of interest** | | | | |
| ***NR3C1***  NM_001018076.1 | Glucocorticoid receptor gene | forward | ttcaaaagagcagtggaagga | 80 |
|  |  | reverse | tttcttcgaattttatcgatgatg |  |
| ***SLC6A4***  NM_001045.5 | Serotonin transporter gene | forward | tgtctgaggtggccaaaga | 68 |
|  |  | reverse | gttggctatcgcttctgcat |  |
| ***FKBP5***  NM_001145777.1 | FKBP Prolyl Isomerase 5 | forward | ccaaacgaaggagcaacagt | 76 |
|  |  | reverse | ccacatctctgcagtcaaaca |  |
| **Housekeeping genes** | | | | |
| ***ACTB***  NM_001101.3 | Actin Beta | forward | ccaaccgcgagaagatga | 97 |
|  |  | reverse | ccagaggcgtacagggatag |  |
| ***B2M***  NM_004048.2 | Beta-2-Microglobulin | forward | ttctggcctggaggctatc | 86 |
|  |  | reverse | tcaggaaatttgactttccattc |  |
| ***GAPDH***  NM_002046.5 | Glyceraldehyde-3-Phosphate Dehydrogenase | forward | agccacatcgctcagacac | 66 |
|  |  | reverse | gcccaatacgaccaaatcc |  |
| ***GUSB***  NM_001293105.1 | Glucuronidase Beta | forward | cgccctgcctatctgtattc | 91 |
|  |  | reverse | tccccacagggagtgtgtag |  |
| ***HPRT1***  NM_000194.2 | Hypoxanthine Phosphoribosyltranferase 1 | forward | tgaccttgatttattttgcatacc | 102 |
|  |  | reverse | cgagcaagacgttcagtcct |  |
| ***PPIA***  NM_021130.4 | Peptidylprolyl Isomerase A | forward | atgctggacccaacacaaat | 97 |
|  |  | reverse | tctttcactttgccaaacacc |  |
| ***RPL13A***  NM_012423.3 | Ribosomal Protein L13a | forward | ggataagaaaccctgcgaca | 91 |
|  |  | reverse | gcctcgaccatcaagcac |  |
| ***18SrRNA***  K03432.1 | 18S ribosomal RNA | forward | ccgattggatggtttagtgag | 88 |
|  |  | reverse | agttcgaccgtcttctcagc |  |
| ***TFRC***  NM_003234.3 | Transferrin Receptor | forward | acctgtccagacaatctccag | 82 |
|  |  | reverse | tgttttccagtcagagggaca |  |

**Supplement figure 2: geNorm analysis of housekeeping genes**

**
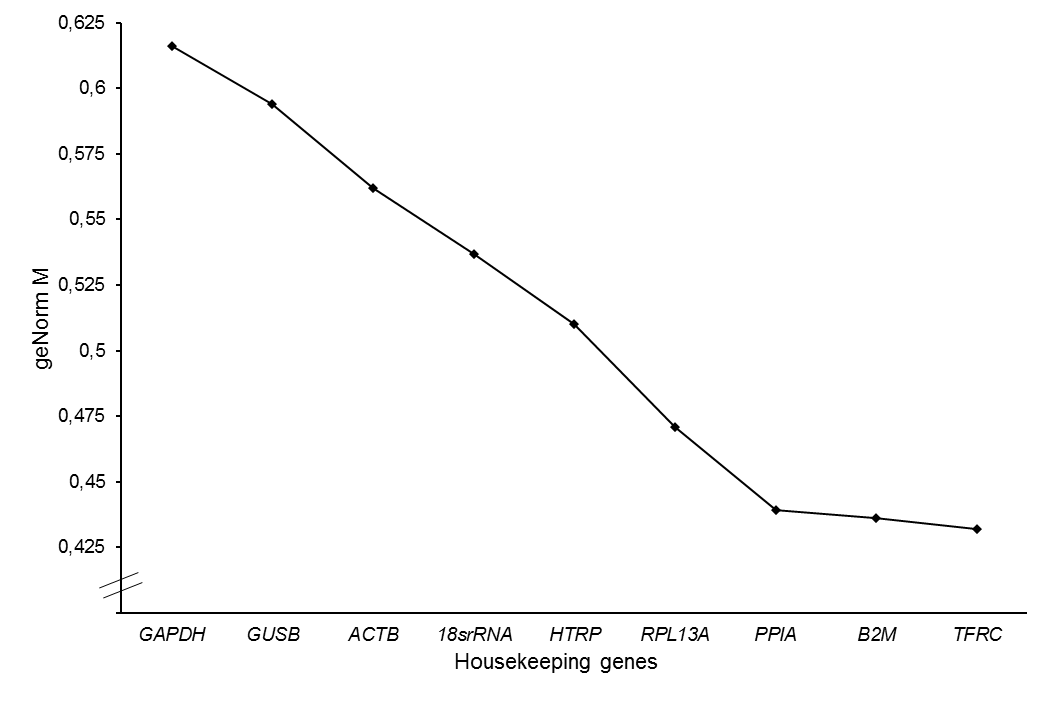
**Note: The chart generated via geNorm method (6) indicates the average expression stability value M of housekeeping genes at each step during stepwise exclusion of the least stable expressed gene. Starting from the least stable gene at the left, the genes are ranked according to increasing expression stability, ending with the three most stable genes to achieve the most accurate normalization on the right (*PPIA*, *B2M*, *TFRC*).

**Supplement 3: Principal Component Analysis**

Given the large amount of CpG sites corresponding to the *SLC6A4* gene, we performed a principal component analysis (PCA) on the covariance structure among the 84 CpG sites using the psych package (7). As previously reported (8), we expected a multi-dimensional structure, possibly including one ‘general methylation factor’, as well as clustering of DNA methylation levels at multiple CpG sites. Therefore, an oblique bi-factor rotation was done after the PCA using the GPArotation package (9, 10). However, results did not largely improve when rotation was applied compared to the PCA without rotation. Analyses suggested a two-component structure for our data (Suppl. Fig. 3). Extracted variance components and their intercorrelations are shown in Supplemental Table 3.


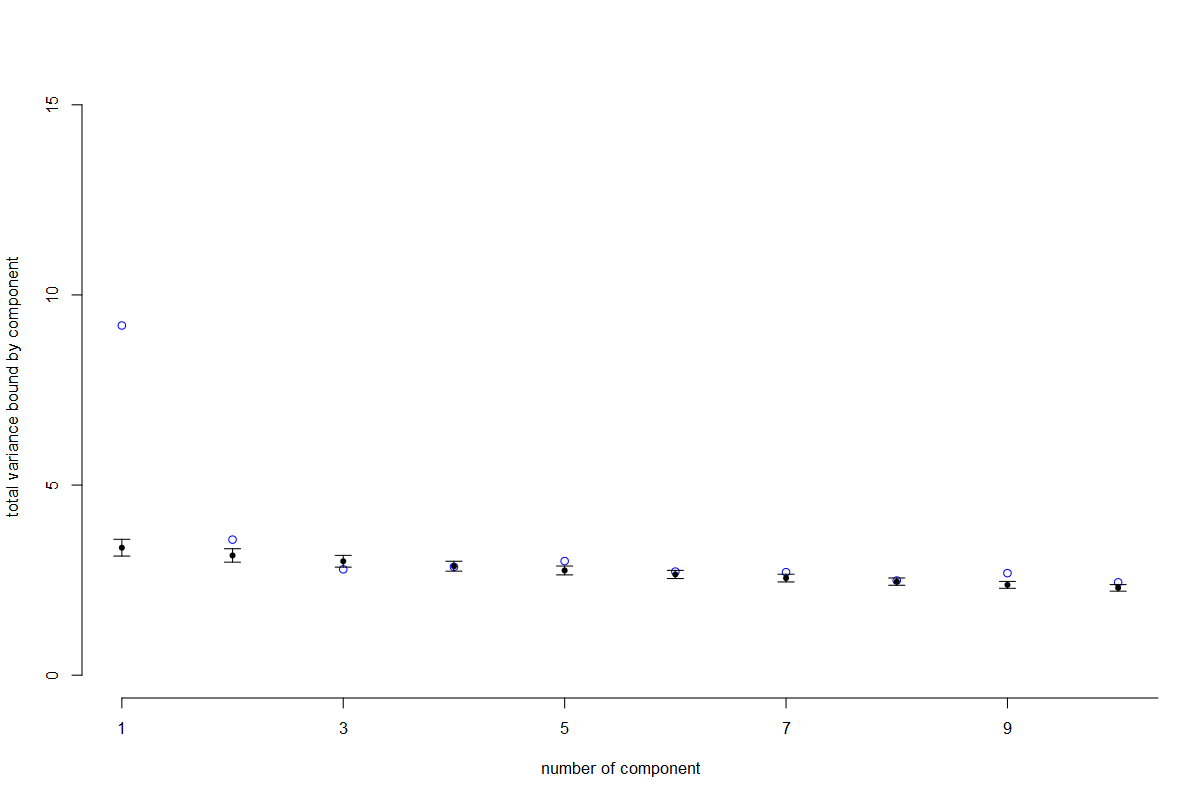
**Supplement figure 3**. Screeplot of the principal component analyses (PCA) of *SLC6A4* DNA methylation data, suggesting a solution of 2 variance components. Blue circles represent the empirically estimated components. Black dots indicate means and 95% confidence intervals of component estimations upon presence of no covariance in between the methylation of all 84 CpG sites.

**Supplement table 3.** Principal component analysis (PCA) on DNA methylation within the *SLC6A4.*

| Principal components | PC1 | PC2 |
| --- | --- | --- |
| Total variance (eigenvalues) | 9.20 | 3.57 |
| Proportion of variance explained | 10.9% | 4.2% |
| Cumulative proportion | 10.9% | 15.2% |
| Correlation | 0.02 |  |

*Note:* Total variance (eigenvalues) = Total variance in methylation of all *SLC6A4* CpGs explained by the respective components. Proportion of variance explained = Proportion of the total variance which is explained by the specific component (eigenvalue/84), as the total variance of 84 CpGs using standardized values equals 84. Cumulative Proportion = Proportional variance summed over the respective components.

**Supplement 4: Identification of potential confounders and moderators**

Initial analyses were conducted to identify associations of mean DNA_M_ (*FKBP5*, *SLC6A4*) and mRNA expression levels (*NR3C1*, *FKBP5*, *SLC6A4*) with demographic, birth- and health-related characteristics (Suppl. Tab. 4). For *FKBP5,* mean DNA_M_ levels were significantly associated with sex (*p* = .035) with females showing higher levels (*M* = 91.87, *SD* = 1.04) than males (*M* = 91.06, *SD* = 1.69). In addition, *FKBP5* mean DNA_M_ levels were negatively correlated with age (*p* < .001), BMI (*p* < .001), and Apgar score (*p* = .005). For *SLC6A4*, mean DNA_M_ levels were significantly associated with sex (*p* = .007) with females showing higher levels (*M* = 5.34, *SD* = 0.7) than males (*M* = 4.99, *SD* = 0.59). In addition, *SLC6A4* mean DNA_M_ levels were positively correlated with age (*p* < .001) and BMI (*p* < .001). *FKBP5* mRNA expression levels were not significantly related to any demographic, birth- and health-related characteristics (all *p* ≥ .07). *SLC6A4* mRNA expression was significantly associated with sex (*p* = .033) with males showing higher levels (*M* = 1.26, *SD* = 0.69) than females (*M* = 0.97, *SD* = 0.44). For *NR3C1,* mRNA expression levels were positively correlated with birth weight (*p* =.009) and birth length (*p* = .041). These variables were included as covariates in the following analyses testing for group differences and associations with DNA_M_ and mRNA expression levels, respectively. Additional analyses were conducted to identify associations of cortisol AUCi and hairC with demographic, birth- and health-related characteristics (Suppl. Tab. 4). Cortisol AUCi levels were not significantly related to any demographic, birth- and health-related characteristics (all *p* ≥ .094). HairC levels were positively correlated with BMI (*p* = .023). This variable was included as covariate in the following analyses testing for associations with hairC.

**Supplement table 4.** Identification of potential confounders in the association with hairC, cortisol stress reactivity, mean DNA_M_ and mRNA expression

|  | HairC | TSST cortisol AUCi | Mean DNA_M_ | | mRNA expression | | | |
| --- | --- | --- | --- | --- | --- | --- | --- | --- |
| Confounder |  |  | *FKBP5* | *SLC6A4* | | *FKBP5* | *NR3C1* | *SLC6A4* |
| Age (y) | .201 | .587 | <.001 | <.001 | | .078 | .654 | .346 |
| Sex | .465 | .568 | .035 | .007 | | .292 | .711 | .033 |
| BMI | .023 | .208 | <.001 | <.001 | | .301 | .873 | .090 |
| Weight (g) | .331 | .236 | .301 | .351 | | .130 | .009 | .437 |
| Length (cm) | .592 | .173 | .436 | .397 | | .373 | .041 | .542 |
| APGAR 5 min | .090 | .094 | .005 | .412 | | .447 | .970 | .245 |
| Length of gestation | .352 | .192 | .089 | .085 | | .994 | .789 | .578 |
| Prenatal stress exposure | .984 | .858 | .336 | .819 | | .588 | .873 | .656 |

*Note.* P values are presented for the relations between potential confounders and outcomes obtained from correlations for continuous variables and ANOVAs for categorical variables. Abbreviations: BMI, body mass index; TSST, Trier Social Stress Test; AUCi, Area under the curve with respect to increase; DNA_M_, DNA methylation.

**Supplement 5:** **No association of DNA_M_ with mRNA expression in stress-associated genes**

We investigated whether mean DNA_M_ of the investigated genes would predict mRNA expression levels of the respective gene. The regression models did not reveal significant effects of mean DNA_M_ on mRNA expression (*FKBP5*: t(100)=–0.91, *p*=.367, β=­–.09; BF_10_=0.301; *SLC6A4*: t(95)=–0.94, *p*=.348, β=–.10; BF_10_=0.252; Suppl. Fig. 4). Comparable results were achieved when controlling for potential confounding effects in the models. Exclusion of outliers in the respective models did not change any of the results. We also tested whether DNA_M_ at specific CpG sites (*FKBP5*, *SLC6A4*) and co-methylated factors (*SLC6A4*) were associated with mRNA expression by replacing mean DNA_M_ by site- and factors-specific DNA_M_ in the regression model. The results were largely comparable and site- as well as factor-specific DNA_M_ did not significantly predict mRNA expression after correction for multiple testing, except for CpG 32 of the *SLC6A4* gene which was positively associated with mRNA expression (chr17:28,562,861; t(95)=3.97, *P*_adj_=.012, β=.37; BF_10_>100). This association also survived when correcting for the covariates sex, age and BMI (t(76)=3.87, *P*_adj_=.019, β=.41). However, DNA_M_ levels at this CpG site show very low variability (*SD*=0.67%) and overall low methylation levels (*M*=2.93%), thus this finding must be treated with caution. Together these findings indicate little to no predictive effect of DNA_M_ on mRNA expression in the stress-associated genes.


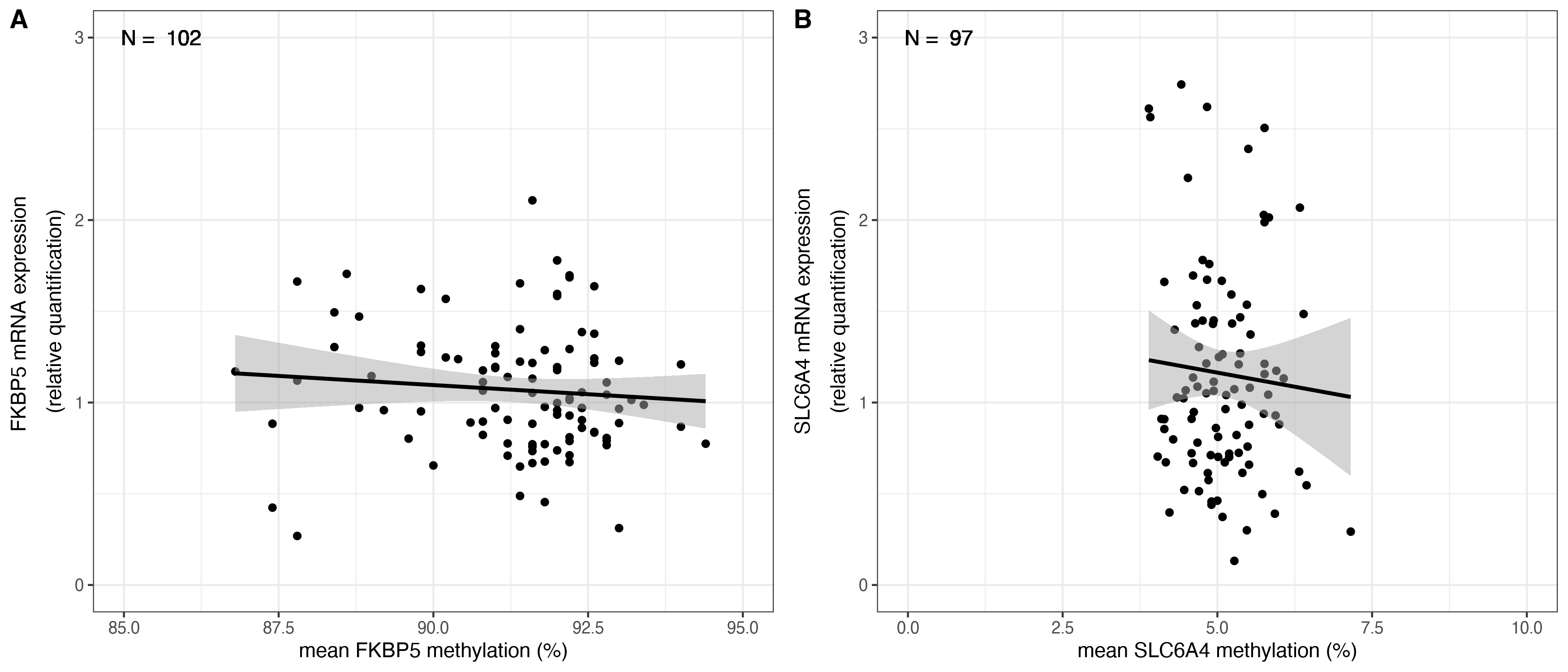


**Supplement figure 4**. Mean DNA_M_ did not statistically affect mRNA expression in stress-associated genes (A: *FKBP5; B:* *SLC6A4;* all *p* ≥ .348)

**Supplement 6: Bayes Factor Analyses**

Bayes factors were calculated for all tested hypotheses to examine the likelihood of the alternative hypothesis (H1) compared to the null hypothesis (H0) given the observed data (11, 12). We used the lmBF function of the BayesFactor package (13) in R with default priors and 10,000 iterations on each model containing predictors of interest without covariates. A Bayes factor above 1 is considered in favour of the alternative hypothesis given the observed data and defined priors, and a common convention is to interpret a Bayes factor ≥ 3 as moderate evidence and ≥ 10 as strong evidence for the H1 (12, 14). Contrarily, a Bayes factor below 1 is considered as evidence for the H0, with values ≤ 1/3 indicating moderate evidence and ≤ 1/10 strong evidence for the H0.

Bayes factor analyses of DNA_M_ at specific CpG sites (five CpGs for *FKBP5*; 84 CpG sites for *SLC6A4*) and two co-methylated factors (*SLC6A4*) largely support findings of the frequentist analyses. For the association between DNA_M_ and mRNA expression of the respective gene, Bayes factor analysis provided moderate evidence for the H0 (BF_10_ < 1/3, i.e., absence of an effect) for 60 of the tests (65.9%) and anecdotal evidence for the H0 (BF_10_ < 1 & > 1/3) for 27 of the tests. Contrarily, there was anecdotal evidence for the alternative hypothesis (BF_10_ > 1 & < 3) for three CpGs within the *SLC6A4* gene (CpG 1, 22, 56) and extreme evidence for the H1 (BF_10_ > 100) for one CpG of the *SLC6A4* gene (CpG 32), which was also identified in the frequentist analysis, providing evidence for an association between DNA_M_ at this CpG site and mRNA expression. Regarding group differences in DNA_M_, Bayes factor analysis revealed similar results, with moderate evidence for the H0 (BF_10_ < 1/3, i.e., absence of an effect) for the majority of the tests (*N* = 59, 66.3%), anecdotal evidence for the H0 for 20 of the tests (BF_10_ < 1 & > 1/3) and strong evidence for the H0 (BF_10_ < 1/10) for seven of the tests. In contrast, there was anecdotal evidence (BF_10_ > 1 & < 3) for group differences in DNA_M_ of two CpGs within the *SLC6A4* gene (CpG 14, 31) and of one CpG within the *FKBP5* gene (CpG 3). Similar results were obtained from the analysis of cortisol levels. Regarding cortisol stress reactivity, we found moderate evidence for the H0 (BF_10_ < 1/3, i.e., absence of an effect) for 66 of the tests (72.5%), anecdotal evidence for the H0 (BF_10_ < 1 & > 1/3) for 22 of the tests. Anecdotal evidence for the H1 (BF_10_ > 1 & < 3) was observed for two CpG sites within the *SLC6A4* gene (CpG 3, 72) and for one CpG site within the *FKBP5* gene (CpG 3). Almost identical results were observed for the analysis of hair cortisol, but with moderate evidence for the H0 (BF_10_ < 1/3, i.e., absence of an effect) for 67 of the tests (73.6%). Furthermore, anecdotal evidence for the alternative hypothesis (BF_10_ > 1 & < 3) was observed only for two CpG sites within the *SLC6A4* gene (CpG 1, 42).

**Supplemental References**

1: Alexander, N., Kirschbaum, C., Wankerl, M., Stauch, B. J., Stalder, T., Steudte-Schmiedgen, S. et al. Glucocorticoid receptor gene methylation moderates the association of childhood trauma and cortisol stress reactivity. *Psychoneuroendocrinology*. 90, 68–75 (2018).

2: McGowan, P. O., Sasaki, A., D’Alessio, A. C., Dymov, S., Labonté, B., Szyf, M. et al. Epigenetic regulation of the glucocorticoid receptor in human brain associates with childhood abuse. *Nat Neurosci*. *12*, 342–348 (2009).

3: Palma-Gudiel, H., Fañanás, L. An integrative review of methylation at the serotonin transporter gene and its dialogue with environmental risk factors, psychopathology and 5-HTTLPR. *Neurosci Biobehav Rev*. 72, 190-209 (2017).

4: Klengel, T., Mehta, D., Anacker, C., Rex-Haffner, M., Pruessner, J. C., Pariante, C. M. et al. Allele-specific FKBP5 DNA demethylation mediates gene–childhood trauma interactions. *Nat Neurosci*. 16, 33-41 (2013).

5: Burns, M., Valdivia, H. Modelling the limit of detection in real-time quantitative PCR. *Eur Food Res Technol. 226*, 1513–1524 (2008).

6: Vandesompele, J., De Preter, K., Pattyn, F., Poppe, B., Van Roy, N., De Paepe, A. et al. Accurate normalization of real-time quantitative RT-PCR data by geometric averaging of multiple internal control genes. *Genome Biol.* 3, 1-12 (2002).

7: Revelle, W. psych: Procedures for Psychological, Psychometric, and Personality Research. Northwestern University, Evanston, Illinois. R package version 2.1.3. <https://CRAN.R-project.org/package=psych> (2021).

8: Wankerl, M., Miller, R., Kirschbaum, C., Hennig, J., Stalder, T., Alexander, N. Effects of genetic and early environmental risk factors for depression on serotonin transporter expression and methylation profiles. *Transl Psychiatry*. 4, e402 (2014).

9: Bernaards, C. A., Coen, A., Jennrich, R. I. Gradient projection algorithms and software for arbitrary rotation criteria in factor analysis. *Educ Psychol Meas.* 65, 676-696 (2005).

10: Jennrich, R. I., Bentler, P. M. Exploratory bi-factor analysis: The oblique case. *Psychometrika.* 77, 442–454 (2012).

11: Jeffreys, H. *The theory of probability*. Oxford University Press (1961).

12: Kass, R. E., Raftery, A. E. Bayes factors. *J Am Stat Assoc.* 90, 773–795 (1995).

13: Morey, R. D., Rouder, J. N., Jamil, T. BayesFactor: Computation of bayes factors for common designs. R package version 0.9.12-4.2. <https://CRAN.R-project.org/package=BayesFactor> (2018).

14: Schönbrodt, F. D., Wagenmakers, E.-J. Bayes factor design analysis: Planning for compelling evidence. *Psychon Bull Rev*. 25, 128–142 (2018).
